# Comparative Clinical Characteristics of ICU and Non-ICU Hospitalized Patients With Human Anaplasmosis

**DOI:** 10.1093/ofid/ofag313

**Published:** 2026-05-21

**Authors:** Igor Dumic, Marie Schultz, Christopher Benny, Jennifer Rich, Aryan Shiari

**Affiliations:** Department of Hospital Medicine, Mayo Clinic Health System, Eau Claire, Wisconsin, USA; Graduate Research Intern Program, Department of Hospital Medicine, Mayo Clinic Health System, Eau Claire, Wisconsin, USA; Medical College of Wisconsin–Central Wisconsin, Wausau, Wisconsin, USA; Department of Practice Analytics, Mayo Clinic Health System, Eau Claire, Wisconsin, USA; Department of Pulmonary and Critical Care Medicine, Mayo Clinic Health System, Eau Claire, Wisconsin, USA

**Keywords:** *Anaplasma phagocytophilum*, anaplasmosis, hyponatremia, intensive care unit, tick-borne disease

## Abstract

**Background:**

Human granulocytic anaplasmosis (HGA) can cause severe illness requiring intensive care, but factors associated with critical illness among hospitalized patients are incompletely described.

**Method:**

We performed a retrospective multicenter cohort study of adults hospitalized with PCR-confirmed HGA at Mayo Clinic Rochester and affiliated Mayo Clinic Health System sites in Minnesota and Wisconsin from 2014 through 2024. Patients who required ICU-level care were compared with those managed on general medical wards using exploratory univariate analyses (Fisher exact and Kruskal–Wallis tests).

**Results:**

Among 101 hospitalized patients, 12 (12%) required ICU-level care. Symptoms documented at hospital presentation/admission were more frequent in ICU patients, including vomiting (64% vs 30%) and abdominal pain (55% vs 26%). On admission, ICU patients had lower sodium (mean 128.8 vs 132.3 mmol/L) and higher total bilirubin (mean 1.3 vs 0.8 mg/dL). Platelet nadir during hospitalization was lower in ICU patients (mean 54.8 × 10^9^/L vs 84.1 × 10^9^/L). Peak ferritin, measured in a subset, was markedly higher in ICU patients (median 21 666 vs 961 µg/L). The composite endpoint of HGA-related complications or serious treatment-related adverse events occurred more often in ICU patients (75% vs 17%); one death occurred in the ICU group.

**Conclusions:**

In hospitalized adults with HGA, ICU-level care was associated with gastrointestinal symptoms at presentation/admission, admission hyponatremia and hyperbilirubinemia, lower platelet nadir, and marked hyperferritinemia when measured. Because these findings were derived from univariate group-level comparisons with substantial overlap between groups, they should be viewed as hypothesis-generating rather than individually predictive or sufficient as stand-alone triage criteria.

## Teaser

Among hospitalized adults with PCR-confirmed HGA, 12% required ICU-level care. Gastrointestinal symptoms at presentation/admission, hyponatremia, hyperbilirubinemia, lower platelet nadir, and marked hyperferritinemia were associated with ICU-level care at the group level but should not be interpreted as individual predictors or triage criteria.

*Anaplasma phagocytophilum* (*A. phagocytophilum*) is an emerging, Gram-negative, obligate intracellular bacterium transmitted to humans by *Ixodes scapularis* in the northeastern and upper Midwestern United States and by *Ixodes pacificus* in the Pacific Northwest [1–3]. The reported incidence of human granulocytic anaplasmosis (HGA) has increased in the United States in parallel with other tick-borne zoonoses transmitted by Ixodes ticks, likely reflecting a combination of climate change, expanding tick exposure, improved diagnostic testing, and increased clinician recognition [4–7]. Similar trends have been described in Europe, where the disease is transmitted by *Ixodes ricinus*, including geographic expansion and increasing hospitalization rates in France [8].

Approximately two-thirds of patients with HGA can be treated with doxycycline in an outpatient setting, as the disease often presents in a mild form. However, about one-third of patients require hospitalization, with older age and immunosuppression being associated with an increased need for inpatient care [2]. In a recent French multicenter cohort, none of the hospitalized patients required admission to an intensive care unit (ICU) [8]; however, an ICU case with acute kidney injury (AKI) and rhabdomyolysis was subsequently reported from the same region [9]. In contrast, in the United States, up to 10% of patients may require treatment in an ICU [1, 2].

Clinical characteristics and presenting features of patients with anaplasmosis requiring ICU admission have not been extensively described in the literature. The primary aim of this retrospective cohort study was to compare clinical presentations and laboratory parameters between patients admitted to the general medical ward and those requiring ICU care, and to evaluate outcomes in these 2 cohorts from a highly endemic region of the United States.

## METHODS

### Study Design and Patient Population

We conducted a retrospective cohort study and chart review of all adult patients diagnosed with *A. phagocytophilum* infection (ICD-10 codes A77.49 or A79.82) who had a positive EDTA whole-blood *A. phagocytophilum* polymerase chain reaction (PCR) test and who were admitted from January 1, 2014, through December 31, 2024, to Mayo Clinic in Rochester, Minnesota, or to affiliated sites within the Mayo Clinic Health System across Wisconsin and Minnesota. Mayo Clinic in Rochester is a large, quaternary care, 1200-bed academic medical center, whereas Mayo Clinic Health System comprises multiple smaller community teaching and critical access hospitals ranging from 30 to 200 beds. All diagnostic testing for tick-borne diseases was performed at Mayo Clinic Laboratories in Rochester, Minnesota, using a whole-blood EDTA real-time Ehrlichia/Anaplasma PCR assay with DNA probe hybridization targeting groEL, ensuring standardized laboratory evaluation across sites. The study was reviewed and approved by the Mayo Clinic Institutional Review Board (18-007901). Minnesota patients who did not provide research authorization in accordance with Minnesota state statute were excluded from the study.

### Study Objectives

The primary objective was to describe patients' demographics, clinical and laboratory characteristics, and outcomes between patients with HGA hospitalized in ICU and non-ICU settings. The secondary objective was to explore clinical and laboratory features associated with ICU-level care.

### Data Collection and Definitions

Eligible patients were identified using ICD-10 diagnosis codes consistent with anaplasmosis (A77.49 or A79.82) based on hospital discharge records. All cases then underwent manual chart review to confirm eligibility, including documentation of a positive blood PCR test for *A. phagocytophilum*. Presenting symptoms reported in Table 2 were abstracted from documentation at initial hospital presentation/admission only. Coinfections were abstracted if documented on admission and were limited to concomitant Ixodes-transmitted infections. Relevant variables were subsequently abstracted from the electronic medical record and entered into research electronic data capture for standardized data management [10, 11].

Laboratory data were abstracted using prespecified time points. For routinely obtained chemistries and counts used to reflect early presentation (eg, sodium and lymphocyte count), the first documented value at hospital presentation/admission was recorded. For selected markers intended to reflect illness severity during hospitalization (eg, platelet count, ferritin, C-reactive protein (CRP), lactate dehydrogenase (LDH), and procalcitonin when available), the extreme value during hospitalization was recorded (nadir for platelet count; peak for ferritin/CRP/LDH/procalcitonin).

Comorbidities were defined as diabetes, cirrhosis, obesity, chronic kidney disease stage III or above (ie, eGFR <60 mL/min/1.73 m^2^), chronic obstructive pulmonary disease, autoimmune disease, or immunosuppression. Tick-borne coinfection was defined as a concomitant Ixodes-transmitted infection documented at presentation/admission rather than a hospital-acquired infection. HGA-related complications were defined as seizure, respiratory failure, myocarditis, shock, hemophagocytic lymphohistiocytosis, AKI, liver failure, hepatitis, or disseminated intravascular coagulation. Serious treatment-related adverse events were abstracted separately. For continuity with the original analysis, Table 4 reports a composite of either an HGA-related complication or a serious treatment-related adverse event; treatment-related events are described separately in the “Results and Discussion.” Shock was abstracted based on clinician documentation and could reflect septic shock attributable to HGA or anaphylactic shock temporally associated with antibiotic exposure.

### Statistical Analysis

Categorical variables were summarized as frequencies and percentages. Continuous variables were summarized as mean (SD) or median (IQR). Groups were compared using Fisher exact tests (categorical variables) or Kruskal–Wallis tests (continuous variables). Given the small number of ICU cases (n = 12), analyses were exploratory and limited to univariate comparisons; multivariable modeling was not performed because estimates would have been unstable. For all tests, the threshold for statistical significance was set at 0.05. Analyses were performed in SAS Enterprise Edition, version 3.82.

## RESULTS

### Study Cohort and Baseline Characteristics

Among 101 hospitalized adults with PCR-confirmed HGA, 12 (12%) required ICU-level care; 40% of the overall cohort were female. The majority of patients were White (97%). Demographic characteristics, including age, sex, ethnicity, and race, did not differ significantly between ICU and non-ICU patients.

A higher proportion of patients in the ICU group had at least 1 comorbidity compared with the non-ICU cohort (92% vs 70%); however, this difference did not reach statistical significance (*P* = .1712). Social factors, including active tobacco use, alcohol use disorder, and active illicit drug use, were infrequently reported and did not differ meaningfully between groups. All documented coinfections were tick-borne infections present on admission. Of 14 coinfected patients, 9 had Lyme disease alone, 2 had *Ehrlichia muris eauclairensis* alone, 2 had concurrent Lyme disease and *Ehrlichia muris eauclairensis*, and 1 had *Babesia microti*. Coinfection was more common in ICU patients (33% vs 11%), although this difference was not statistically significant (*P* = .0603) (Table 1).

**Table 1.** Demographics and Comorbidities

	General Medical Ward (N = 89)	ICU (N = 12)	Total (N = 101)	*P* Value
Age at admission, y				.7767^a^
Mean (SD)	69.5 (11.51)	70.0 (13.64)	69.5 (11.71)	
Median (IQR)	72.0 (64.0, 77.0)	72.5 (61.0, 80.0)	72.0 (64.0, 77.0)	
Gender, n (%)				.7572^b^
Female	37 (41.6%)	4 (33.3%)	41 (40.6%)	
Male	52 (58.4%)	8 (66.7%)	60 (59.4%)	
Ethnicity, n (%)				1.0000^b^
Central American	1 (1.1%)	0 (0.0%)	1 (1.0%)	
Not Hispanic or Latino	87 (97.8%)	12 (100.0%)	99 (98.0%)	
Other Spanish culture	1 (1.1%)	0 (0.0%)	1 (1.0%)	
Race, n (%)				1.0000^b^
American Indian/Alaskan Native	2 (2.2%)	0 (0.0%)	2 (2.0%)	
White	86 (96.6%)	12 (100.0%)	98 (97.0%)	
Asian	1 (1.1%)	0 (0.0%)	1 (1.0%)	
Comorbidities, n (%)	61 (70.1%)	11 (91.7%)	72 (72.7%)	.1712^b^
Social habits				
Active smoking, n (%)	8 (9.1%)	1 (8.3%)	9 (9.0%)	1.0000^b^
Alcohol use disorder, n (%)	1 (1.1%)	1 (8.3%)	2 (2.0%)	.2267^b^
Active illicit drug use, n (%)	1 (1.2%)	0 (0.0%)	1 (1.1%)	1.0000^b^
History of tick bite within 3 m, n (%)	31 (36.0%)	2 (20.0%)	33 (34.4%)	.4859^b^
Documented tick-borne coinfection, n (%)	10 (11.2%)	4 (33.3%)	14 (13.9%)	.0603^b^

^a^Kruskal–Wallis *P* value.

^b^Fisher exact *P* value.

All documented coinfections were tick-borne infections present on admission and included Lyme disease (n = 9), *Ehrlichia muris eauclairensis* (n = 2), concurrent Lyme disease and *Ehrlichia muris eauclairensis* (n = 2), and *Babesia microti* (n = 1).

### Clinical Presentation

Symptoms documented at hospital presentation/admission are summarized in Table 2.

**Table 2.** Symptoms at Hospital Presentation/Admission

	General Medical Ward (N = 89)	ICU (N = 12)	Total (N = 101)	*P* Value
Fever, n (%)	70 (79.5%)	7 (58.3%)	77 (77.0%)	.1401^a^
Fatigue, n (%)	69 (78.4%)	7 (63.6%)	76 (76.8%)	.2750^a^
Chills/rigors, n (%)	54 (61.4%)	3 (27.3%)	57 (57.6%)	.0496^a^
Nausea, n (%)	40 (45.5%)	6 (54.5%)	46 (46.5%)	.7503^a^
Headache, n (%)	41 (46.6%)	4 (36.4%)	45 (45.5%)	.7497^a^
Myalgia, n (%)	37 (42.5%)	8 (66.7%)	45 (45.5%)	.1337^a^
Cough, n (%)	36 (40.9%)	5 (45.5%)	41 (41.4%)	.7584^a^
Dyspnea, n (%)	29 (33.0%)	7 (58.3%)	36 (36.0%)	.1118^a^
Sweating, n (%)	32 (36.4%)	1 (9.1%)	33 (33.3%)	.0939^a^
Vomiting, n (%)	26 (29.5%)	7 (63.6%)	33 (33.3%)	.0387^a^
Abdominal pain, n (%)	22 (25.6%)	6 (54.5%)	28 (28.9%)	.0530^a^
Confusion, n (%)	23 (25.8%)	6 (50.0%)	29 (28.7%)	.0977^a^
Diarrhea, n (%)	25 (28.4%)	2 (18.2%)	27 (27.3%)	.7223^a^
Arthralgia, n (%)	22 (25.0%)	1 (9.1%)	23 (23.2%)	.4491^a^
Rash, n (%)	4 (4.5%)	1 (8.3%)	5 (5.0%)	.4797^a^

^a^Fisher exact *P* value.

Symptoms reflect documentation at initial hospital presentation/admission. Symptom denominators vary because symptoms were counted as present only when explicitly documented; undocumented symptoms were treated as missing (not absent). Percentages are calculated using available documentation.

Chills/rigors were reported less frequently among patients requiring ICU care compared with those in the non-ICU group (27% vs 61%; *P* = .0496). In contrast, vomiting was more common in ICU patients (64% vs 30%; *P* = .0387), as was abdominal pain (55% vs 26%; *P* = .0530). Rash, an atypical manifestation of anaplasmosis, was documented in 5% of patients overall, including 1 patient in the ICU group and 4 in the non-ICU group.

### Laboratory Characteristics

Laboratory parameters for hospitalized patients with anaplasmosis are summarized in Table 3. The most clinically notable between-group differences involved hyponatremia, hyperbilirubinemia, thrombocytopenia, AKI, and peak ferritin levels.

**Table 3.** Laboratory Values at Presentation and Peak/Nadir During Hospitalization

	General Medical Ward (N = 89)	ICU (N = 12)	Total (N = 101)	*P* Value
Blood counts				
Leukopenia, n (%)	25 (28.1%)	3 (25.0%)	28 (27.7%)	1.0000^a^
Platelet nadir during hospitalization				.0595^b^
N	89	12	101	
Mean (SD)	84.1 (68.87)	54.8 (42.26)	80.6 (66.79)	
Median (IQR)	70 (35, 103)	35 (22, 86)	68 (34, 103)	
Lymphocyte count at presentation				.9732^b^
N	83	12	95	
Mean (SD)	0.8 (0.71)	0.9 (0.78)	0.8 (0.72)	
Median (IQR)	1 (0, 1)	1 (0, 1)	1 (0, 1)	
Blood chemistries				
AKI present at admission/diagnosis, n (%)	15 (16.9%)	5 (41.7%)	20 (19.8%)	.0576^a^
Sodium at presentation				.0161^b^
N	89	12	101	
Mean (SD)	132.3 (4.06)	128.8 (4.53)	131.9 (4.24)	
Median (IQR)	133 (129, 135)	131 (127, 132)	133 (129, 135)	
AST at presentation				.0746^b^
N	85	11	96	
Mean (SD)	112.0 (150.98)	316.6 (591.69)	135.4 (247.59)	
Median (IQR)	74 (39, 120)	111 (57, 288)	75 (40, 130)	
ALT at presentation				.6458^b^
N	84	11	95	
Mean (SD)	97.0 (123.90)	99.0 (114.88)	97.2 (122.31)	
Median (IQR)	50 (33, 102)	50 (40, 83)	50 (33, 102)	
ALP at presentation				.4444^b^
N	83	11	94	
Mean (SD)	122.4 (78.91)	102.2 (46.27)	120.1 (75.92)	
Median (IQR)	105 (77, 141)	96 (75, 118)	104 (76, 141)	
Total bilirubin at presentation				.0135^b^
N	84	11	95	
Mean (SD)	0.8 (0.51)	1.3 (0.80)	0.9 (0.57)	
Median (IQR)	1 (1, 1)	1 (1, 2)	1 (1, 1)	
Inflammatory markers				
Peak CRP during hospitalization				.4315^b^
N	57	4	61	
Mean (SD)	120.6 (77.84)	144.9 (53.50)	122.2 (76.39)	
Median (IQR)	108 (52, 176)	147 (102, 188)	111 (52, 176)	
Peak procalcitonin during hospitalization				.1101^b^
N	20	3	23	
Mean (SD)	3.4 (6.02)	12.7 (17.60)	4.6 (8.36)	
Median (IQR)	1 (0, 2)	4 (1, 33)	1 (1, 4)	
Peak LDH during hospitalization				.1451^b^
N	19	5	24	
Mean (SD)	373.8 (156.11)	575.0 (321.66)	415.7 (209.84)	
Median (IQR)	349 (257, 481)	511 (448, 525)	359 (275, 516)	
Peak ferritin during hospitalization				.0308^b^
N	9	4	13	
Mean (SD)	2834.9 (3525.39)	36 141.0 (43 830.20)	13 082.9 (27 286.42)	
Median (IQR)	961 (781, 4218)	21 666 (4742, 67540)	3552 (904, 5932)	

^a^Fisher exact *P* value.

^b^Kruskal–Wallis *P* value.

AKI, acute kidney injury; AST, aspartate aminotransferase; ALT, alanine aminotransferase; ALP, alkaline phosphatase; CRP, C-reactive protein; LDH, lactate dehydrogenase. Presentation/admission values represent the first recorded laboratory result at hospital presentation. Peak/nadir values represent the extreme value recorded during the index hospitalization (peak for ferritin/CRP/LDH/procalcitonin; nadir for platelet count). Leukopenia was defined as WBC <4 × 10^9^/L.

Sodium at presentation was lower in ICU patients compared with non-ICU patients (mean 128.8 vs 132.3 mmol/L; *P* = .0161), whereas total bilirubin was higher in the ICU group (mean 1.3 vs 0.8 mg/dL; *P* = .0135). Platelet nadir during hospitalization trended lower among ICU patients (mean 54.8 × 10^9^/L vs 84.1 × 10^9^/L; *P* = .0595). AKI present at admission or diagnosis was more frequent in ICU patients than in non-ICU patients (41.7% vs 16.9%; *P* = .0576).

Peak ferritin during hospitalization, available for a limited subset of patients, was markedly higher in ICU patients (median 21 666 vs 961 µg/L; *P* = .0308). CRP was measured in 61 of 101 patients (60.4%), procalcitonin in 23 of 101 patients (22.8%), and lactate dehydrogenase (LDH) in 24 of 101 patients (23.8%). Although absolute values of these inflammatory markers did not differ significantly between ICU and non-ICU patients, values were elevated in the patients in whom they were obtained.

### Complications

ICU patients were more than 4 times as likely to experience the composite endpoint of an HGA-related complication or serious treatment-related adverse event compared with medical ward patients (75.0% vs 17.0%; *P* < .0001). Among ICU patients, complications included shock (n = 6), AKI (n = 5), acute hypoxemic respiratory failure (n = 3), myocarditis (n = 1), hemophagocytic lymphohistiocytosis (n = 1), disseminated intravascular coagulation (n = 1), and rhabdomyolysis (n = 1), with several patients experiencing more than 1 complication.

Two ICU patients developed suspected anaphylactic shock after initiation of empiric antibiotics during the index hospitalization (one after doxycycline and one after ceftriaxone). These episodes were considered treatment-related adverse events rather than direct manifestations of HGA but remained part of the prespecified composite endpoint reported in Table 4.

**Table 4.** Treatment and Outcomes

	General Medical Ward (N = 89)	ICU (N = 12)	Total (N = 101)	*P* Value
Composite complication/adverse event, n (%)	15 (17.0%)	9 (75.0%)	24 (24.0%)	<.0001^a^
Treatment duration, d				.4864^b^
N	89	11	100	
Mean (SD)	12.1 (3.67)	12.1 (4.66)	12.1 (3.76)	
Median (IQR)	10.0 (10.0, 14.0)	14.0 (10.0, 14.0)	10.0 (10.0, 14.0)	
Appropriate empiric therapy, n (%)	81 (92.0%)	11 (91.7%)	92 (92.0%)	1.0000^a^
Outcome, n (%)				.1655^b^
Improved	89 (100%)	11 (91.7%)	100 (99%)	
Death	0 (0.0%)	1 (8.3%)	1 (1.0%)	

^a^Fisher exact *P* value.

^b^Kruskal–Wallis *P* value.

Treatment duration refers to doxycycline therapy. The composite endpoint includes HGA-related complications and rare serious treatment-related adverse events (eg, antibiotic-associated anaphylaxis). Appropriate empiric therapy percentages are based on available data.

### Treatment and Outcomes

There were no statistically significant differences between ICU and non-ICU patients with respect to treatment duration, treatment effectiveness, or overall outcomes. Appropriate empiric antimicrobial therapy was documented in 11 of 12 ICU patients (91.7%) and 81 of 88 non-ICU patients with available data (92.0%). All but 1 patient, who developed an anaphylactic reaction to doxycycline, were ultimately treated with doxycycline for 2–4 weeks. The single patient with doxycycline hypersensitivity was successfully treated with rifampin.

One death occurred in an ICU patient who was immunosuppressed because of chemotherapy and developed acute pulmonary embolism, iliopsoas myositis with severe rhabdomyolysis (creatine kinase >70 000 U/L), and AKI requiring continuous renal replacement therapy. Initial empiric therapy did not include doxycycline, which was added only after the diagnosis of HGA was established.

## DISCUSSION

This retrospective multicenter study evaluated 101 hospitalized adults with HGA over a 10-year period in hyperendemic regions of the United States. Overall, 12% of hospitalized patients required ICU-level care and the mortality rate was 1%. ICU-level care was associated with more frequent gastrointestinal symptoms at presentation, admission hyponatremia and hyperbilirubinemia, lower platelet nadirs, and markedly elevated ferritin levels when measured. However, these findings arose from univariate group-level comparisons. Because vomiting and abdominal pain were also common in non-ICU patients and sodium and bilirubin values overlapped substantially between groups, these features should be interpreted as hypothesis-generating associations rather than individually predictive markers of ICU need.

### Demographics and Comorbidities

Previous studies have suggested that older patients are more likely to present with severe anaplasmosis [2, 4]. In our cohort, no statistically significant difference in mean age was observed between ICU and non-ICU patients. Nevertheless, the overall mean age of 69.5 years aligns with prior reports indicating that hospitalization for HGA is highest among individuals older than 60 years [1]. A recent study found that hospitalized patients had more comorbidities [2], and the Centers for Disease Control and Prevention guide to rickettsial diseases lists comorbidities as predictors of anaplasmosis severity [1]. However, we did not find a statistically significant association between comorbidities and ICU admission, which may reflect limited power in this hospitalized-only cohort.

### Clinical Symptoms and Laboratory Findings on Admission

Gastrointestinal symptoms, including vomiting and abdominal pain documented at hospital presentation, were more common in ICU patients, along with laboratory abnormalities such as elevated bilirubin, hyponatremia, and AKI. These findings may reflect greater early systemic illness and, in some patients, reduced oral intake or volume depletion. Nonetheless, because these symptoms were also present in a substantial minority of ward patients, they should not be viewed as stand-alone indicators or triage criteria for severe disease.

Consistent with previous reports [1–5, 12, 13], fever and nonspecific symptoms—fatigue/malaise, rigors, and chills—were the most common presentations in hospitalized HGA patients. Rash occurred in 5% of patients, aligning with CDC data (<10%) [1] and prior institutional reports (7.7%) [2]. Other series report rash in 7.7%–15% of cases [4, 12], confirming it as an uncommon feature. Compared with human monocytic ehrlichiosis, patients with anaplasmosis are less likely to present with rash [12].

Hyponatremia, though less consistently highlighted in prior HGA cohorts, is well described in other infectious syndromes [14–16]. Hyperbilirubinemia likewise tracks illness severity in several acute infections [17–19]. In our cohort, ICU patients had lower sodium and higher total bilirubin on admission, but the ranges overlapped considerably between groups. Moreover, direct and indirect bilirubin fractions, hemolysis markers, and hepatobiliary imaging were not systematically available, so we could not determine whether hyperbilirubinemia reflected intrahepatic cholestasis/hepatocellular injury, hemolysis, biliary obstruction, or mixed mechanisms.

In our study, all hospitalized patients had thrombocytopenia, a well-recognized feature of HGA. Prior systematic reviews and cohort studies, including both hospitalized and outpatient cases, report thrombocytopenia in 60%–80% of patients [2, 4, 5, 12]. The higher prevalence in our cohort likely reflects inclusion of only hospitalized patients, who generally have more severe illnesses. Similar findings were reported in hospitalized patients in France, where over 90% exhibited thrombocytopenia. ICU patients had more pronounced thrombocytopenia and lower platelet nadirs than non-ICU patients, although this did not appear to affect outcomes. In contrast, thrombocytopenia is less common in children [20], reflecting generally milder disease in the pediatric population.

AKI affected 20% of patients in our cohort and occurred more frequently in ICU patients (41.7% vs 16.9%; *P* = .0576). A retrospective study from Hershey, Pennsylvania, reported AKI in 24% of HGA patients [4], and the recent French multicenter cohort identified AKI as the most frequent complication [8]. The subsequent French ICU case report underscores that rhabdomyolysis can accompany severe HGA and may contribute to kidney injury [9]. In our cohort, 1 patient who died had severe rhabdomyolysis and AKI requiring continuous renal replacement therapy. Because urine studies, fractional indices, and CK measurements were not uniformly available across the cohort, we could not reliably distinguish prerenal azotemia from intrinsic renal injury or rhabdomyolysis-associated AKI in all cases.

Ferritin is an acute-phase reactant that can rise markedly in inflammatory and hyperinflammatory syndromes [21–23]. In our cohort, peak ferritin was obtained in only a subset of patients and was substantially higher among those requiring ICU-level care; the ICU group had a median peak ferritin of 21 666 µg/L (IQR 4742–67 540), compared with 961 µg/L (IQR 781–4218) in non-ICU patients (Table 3). Because ferritin testing was not uniform and may have been preferentially obtained in sicker patients, this observation should be interpreted as hypothesis-generating. Higher ferritin levels also raise the possibility that secondary HLH may have been underrecognized. Only 1 ICU patient carried a clinical diagnosis of HLH, although *A. phagocytophilum* is a recognized infectious trigger of secondary HLH [24–26]. Retrospective HScore calculation was not feasible because several components (including triglycerides, fibrinogen, organomegaly, and bone marrow findings) were not uniformly available. In practice, markedly elevated or rising ferritin despite appropriate doxycycline should prompt evaluation for secondary HLH.

### Level of Care and Prior Literature

Earlier HGA studies often combined hospitalized and outpatient-treated patients. While recent work has highlighted differences between these groups [2], few studies have specifically compared ICU and non-ICU admissions. The recent French multicenter cohort reported no ICU admissions [8], but the subsequent French ICU case demonstrates that critical illness does occur in Europe [9]. Differences across cohorts may reflect case mix, timing of diagnosis, referral patterns, and possibly pathogen or host factors, but these explanations remain speculative [5, 12, 13, 27]. In children, only 7% required hospitalization and none required ICU care, supporting generally milder pediatric disease [20].

### Coinfections

Coinfections among pathogens transmitted by Ixodes ticks are well recognized. In this study, all documented coinfections were tick-borne infections present on admission. Of 14 coinfected patients, 9 had Lyme disease alone, 2 had *Ehrlichia muris eauclairensis* alone, 2 had concurrent Lyme disease and *Ehrlichia muris eauclairensis*, and 1 had *Babesia microti*. We did not identify a statistically significant association between coinfection and ICU admission, although coinfection was numerically more common in ICU patients (33% vs 11%). Given the small number of coinfected ICU patients, this finding should be interpreted cautiously. Prior studies similarly identify Lyme disease as the most common coinfection in patients with anaplasmosis [2, 8, 20, 28].

### Complications and Mortality

In this study, ICU patients were approximately 4 times more likely to meet the composite complication endpoint during hospitalization. Among ICU patients, complications included shock, respiratory failure, AKI, myocarditis, HLH, disseminated intravascular coagulation, and rhabdomyolysis, with several patients experiencing more than one complication. Two patients also developed suspected antibiotic-associated anaphylactic shock; these events were temporally related to empiric therapy and were not considered direct manifestations of HGA.

The aggregate complication rate in our cohort (24%) was modestly lower than that reported in comparable retrospective studies from France (33%) and the United States (37.4%) [2, 8]. Because our composite outcome also captured rare severe treatment-related adverse events, direct comparisons across studies should be interpreted cautiously. In contrast, systematic reviews generally report somewhat higher rates of complications and mortality [5, 12], likely reflecting publication and selection bias, as more severe or atypical cases are disproportionately represented in published reports. In one such review, complications were documented in 40.5% of cases overall [12].

Unlike babesiosis, which has been associated with acute liver failure [29], acute liver failure has not been reported in human HGA despite hepatitis being a common manifestation. Compared with human monocytic ehrlichiosis, HGA appears to be associated with fewer complications [13]. Splenectomy may also represent an underrecognized risk factor for severe anaplasmosis and multiorgan failure [30].

### Treatment and Outcomes

Treatment approaches and overall outcomes were generally similar between ICU and non-ICU patients. Most patients in both groups responded favorably to antimicrobial therapy and recovered clinically. In 2 patients with suspected Anaplasma-associated meningoencephalitis and sepsis, the treating clinicians administered an initial intravenous dose of doxycycline 200 mg to potentially enhance central nervous system penetration. Although this approach may appear conceptually compelling, it has not been systematically studied and is not currently recommended by existing clinical guidelines, even in the presence of shock.

One death occurred among ICU admissions (1% mortality), and the cause was multifactorial. The patient was immunosuppressed because of chemotherapy and had acute pulmonary embolism, severe iliopsoas myositis with negative cultures, rhabdomyolysis, and AKI. Initial empiric antimicrobial therapy consisted of cefepime and vancomycin; doxycycline was added only after HGA was diagnosed. Delayed antianaplasma therapy may have contributed to the poor outcome, similar to other reported fatal cases in which appropriate treatment was not started promptly [7, 31]. The mortality rate for this cohort is comparable to prior reports [1, 5] but lower than that described in some systematic reviews [7, 12, 13], which may reflect publication and selection bias of the latter. Additionally, because this cohort was derived from highly endemic regions where clinicians have substantial experience managing anaplasmosis and other tick-borne infections, the low observed mortality may reflect heightened clinical recognition and more timely initiation of empiric antimicrobial therapy. However, even in endemic regions, disease can cause diagnostic challenges and go underrecognized, resulting in fatalities [31].

### Strengths and Limitations

This study has several notable strengths. To our knowledge, it is the first investigation to directly compare patients with anaplasmosis requiring ICU-level care with those managed in non-ICU settings and to systematically evaluate factors associated with ICU admission. Although the absolute number of critically ill patients was limited, this cohort represents one of the largest reported to date of hospitalized patients with anaplasmosis, spanning a 10-year period across 2 hyperendemic regions of the United States. The inclusion of patients from both a large academic quaternary care center and community hospitals in rural Wisconsin and Minnesota enhances the external validity and generalizability of the findings. Finally, diagnostic rigor strengthens the study, as only definite cases confirmed by blood PCR were included, reducing misclassification bias and ensuring a high degree of diagnostic accuracy.

This study has several limitations. First, the overall sample size was relatively small, particularly with respect to critically ill patients, as only 12 individuals required ICU-level care. The limited number of ICU admissions reduces statistical power, increases the risk of type II error, and may limit the ability to detect clinically meaningful differences between groups. Additionally, given the limited number of ICU cases and multiple unadjusted comparisons, *P* values near the threshold of statistical significance should be interpreted cautiously. The small ICU sample also precluded reliable multivariable analysis; consequently, residual confounding is likely and no independent predictors of ICU-level care can be inferred from these data.

Second, the retrospective design introduces inherent limitations. Data collection relied on electronic medical record documentation, which may have resulted in incomplete or inconsistent capture of symptoms, comorbidities, laboratory testing, and timing of treatment initiation. Not all laboratory markers, including ferritin, CRP, procalcitonin, bilirubin fractions, hemolysis studies, creatine kinase, urinalysis, or urine indices, were obtained uniformly across the cohort, limiting mechanistic interpretation of hyperbilirubinemia and renal involvement. The composite complication endpoint also captured rare severe treatment-related adverse events (eg, antibiotic-associated anaphylaxis), which may modestly overestimate complication rates when interpreted strictly as manifestations of HGA. Finally, the study population was drawn from hyperendemic regions in Wisconsin and Minnesota, and the majority of patients were White, which may limit generalizability.

In summary, among hospitalized adults with HGA, ICU-level care was associated with more frequent gastrointestinal symptoms at presentation/admission, admission hyponatremia and hyperbilirubinemia, lower platelet nadirs, and marked hyperferritinemia when measured. These findings better characterize severe hospitalized HGA at the group level, but because of overlap between groups and the limited sample size, they should not be used as individual predictors or triage criteria.

### Patient Consent Statement

The study was reviewed and approved by the Mayo Clinic Institutional Review Board (IRB #18-007901). The requirement for individual informed consent was waived due to the retrospective nature of the study and use of deidentified data. The study was conducted in accordance with the ethical standards of the institutional research committee and with the principles of the Declaration of Helsinki.
